# Supplementary material for: The Impact of a Video-Educational and Tele-Supporting Program on the Caregiver–Stroke Survivor Dyad During Transitional Care (D-STEPS: Dyadic Support Through Tele-Health and Educational Programs in Stroke Care): A Longitudinal Study Protocol
Source: Healthcare (Basel). 2025 Aug 18;13(16):2039. doi: 10.3390/healthcare13162039 (PMC12385329; doi:10.3390/healthcare13162039)
Supplement: Supplementary file 1 [file healthcare-13-02039-s001.zip › healthcare-3778691-supplementary.pdf]

healthcare

MDPI

Supplementary Materials 1: TIDieR (Template for Intervention Description and Replication) Checklist.

TIDieR
Template for Intervention Description and Replication

The TIDieR (Template for Intervention Description and Replication) Checklist*:
Information to include when describing an intervention and the location of the information

Item number	Item	Where located **	
		Primary paper (page or appendix number)	Other † (details)
	**BRIEF NAME**	X (p.1)	
**1.**	Provide the name or a phrase that describes the intervention.	____________	______________
	**WHY**	X(p.2-3)	
**2.**	Describe any rationale, theory, or goal of the elements essential to the intervention.	____________	______________
	**WHAT**	X(p. 3-4)	
**3.**	Materials: Describe any physical or informational materials used in the intervention, including those provided to participants or used in intervention delivery or in training of intervention providers. Provide information on where the materials can be accessed (e.g. online appendix, URL).	____________<br>X (p. 4-5-6-7-8)	______________
**4.**	Procedures: Describe each of the procedures, activities, and/or processes used in the intervention, including any enabling or support activities.	____________	______________
	**WHO PROVIDED**	X(p.5- 6-7-8-9)	
**5.**	For each category of intervention provider (e.g. psychologist, nursing assistant), describe their expertise, background and any specific training given.	____________	______________
	**HOW**	X(p.7-8-9-10-11)	
**6.**	Describe the modes of delivery (e.g. face-to-face or by some other mechanism, such as internet or telephone) of the intervention and whether it was provided individually or in a group.	____________	______________
	**WHERE**	X(p.7-8-9-10-11)	
**7.**	Describe the type(s) of location(s) where the intervention occurred, including any necessary infrastructure or relevant features.	____________	______________
	**WHEN and HOW MUCH**		
**8.**	Describe the number of times the intervention was delivered and over what period of time including the number of sessions, their schedule, and their duration, intensity or dose.	X(p.7-8-9-10-11)	______________
	**TAILORING**		
**9.**	If the intervention was planned to be personalised, titrated or adapted, then describe what, why, when, and how.	X (p.7-8-9)	______________
	**MODIFICATIONS**		
**10.‡**	If the intervention was modified during the course of the study, describe the changes (what, why, when, and how).	no	______________
	**HOW WELL**		

**11.**	Planned: If intervention adherence or fidelity was assessed, describe how and by whom, and if any strategies were used to maintain or improve fidelity, describe them.	X(p.7-8-9-10-11-12)	______________
**12.ǂ**	Actual: If intervention adherence or fidelity was assessed, describe the extent to which the intervention was delivered as planned.	X(p.9-10-11-12)	______________

** **Authors** - use N/A if an item is not applicable for the intervention being described. **Reviewers** – use '?' if information about the element is not reported/not sufficiently reported. .

† If the information is not provided in the primary paper, give details of where this information is available. This may include locations such as a published protocol or other published papers (provide citation details) or a website (provide the URL).

ǂ If completing the TIDieR checklist for a protocol, these items are not relevant to the protocol and cannot be described until the study is complete.

* We strongly recommend using this checklist in conjunction with the TIDieR guide (see *BMJ* 2014;348:g1687) which contains an explanation and elaboration for each item.

* The focus of TIDieR is on reporting details of the intervention elements (and where relevant, comparison elements) of a study. Other elements and methodological features of studies are covered by other reporting statements and checklists and have not been duplicated as part of the TIDieR checklist. When a **randomised trial** is being reported, the TIDieR checklist should be used in conjunction with the CONSORT statement (see www.consort-statement.org) as an extension of **Item 5 of the CONSORT 2010 Statement.** When a **clinical trial protocol** is being reported, the TIDieR checklist should be used in conjunction with the SPIRIT statement as an extension of **Item 11 of the SPIRIT 2013 Statement** (see www.spirit-statement.org). For alternate study designs, TIDieR can be used in conjunction with the appropriate checklist for that study design (see www.equator-network.org).

Supplementary Materials 2: Step-by-step guide to accessing digital materials via computer or laptop (Italian and English versions)

Brochure Educativa – Mobilizzazione Precoce / Early Mobilization

Versione Italiana

COSA SERVE:

- Un computer (PC o portatile) con casse o cuffie
- Connessione Internet (se si usano i video via email)
- Una Pennetta USB (se consegnata dall'infermiere)
- Un'email personale (se i video sono inviati digitalmente)

OPZIONE 1: GUARDARE I VIDEO DALLA CHIAVETTA USB

1. Accendere il computer.
2. Inserire la pennetta USB nella porta USB del PC.
3. Aprire la cartella della pennetta se non si apre automaticamente.
4. Fare doppio clic sul file video per iniziare a guardare.
5. Usare i tasti volume o le cuffie per ascoltare meglio.
6. Guardare i video più volte, quando serve.

OPZIONE 2: GUARDARE I VIDEO TRAMITE EMAIL

1. Accendere il computer e aprire il browser.
2. Accedere alla propria email.
3. Cercare l'email del nursing navigator.
4. Aprire l'email e cliccare sui link video o allegati.
5. I video si apriranno automaticamente nel browser.
6. Guardarli con calma e ripeterli al bisogno.

INFORMAZIONI IMPORTANTI:

- I video supportano il caregiver.
- Mostrano situazioni anche gravi per aumentare la preparazione.
- Per dubbi, contattare l'infermiere di riferimento.

CONTATTI:

Nursing Navigator: nursingnavigator@dsteps.org | +39 320 456 7890

English Version

WHAT YOU NEED:

- A computer or laptop with speakers or headphones
- Internet connection (for email access)
- USB stick (if provided by the nurse)
- Personal email (for receiving videos via email)

OPTION 1: WATCHING VIDEOS FROM USB

1. Turn on the computer.
2. Insert the USB into the USB port.
3. Open the USB folder if it doesn't open automatically.
4. Double-click the video file to play.
5. Use volume buttons or headphones for better sound.
6. Rewatch as needed.

OPTION 2: WATCHING VIA EMAIL

1. Turn on the computer and open the browser.
2. Log in to your email account.
3. Find the email from the nursing navigator.
4. Click the video links or attachments.
5. Videos will open in the browser.
6. Watch calmly and as often as needed.

KEY INFORMATION:

- Videos support the caregiver.
- Include severe-case examples to improve readiness.
- Contact the nurse if anything is unclear.

CONTACT:

Nursing Navigator: nursingnavigator@dsteps.org I +39 320 456 7890
